# Transcriptome analysis of *Aeromonas hydrophila* infected hybrid sturgeon (*Huso dauricus*×*Acipenser schrenckii*)

**DOI:** 10.1038/s41598-018-36376-2

**Published:** 2018-12-18

**Authors:** Nan Jiang, Yuding Fan, Yong Zhou, Weiling Wang, Jie Ma, Lingbing Zeng

**Affiliations:** Division of Fish Disease, Yangtze River Fisheries Research Institute, Chinese Academy of Fishery Sciences, Wuhan, Hubei 430223 P. R. China

## Abstract

The hybrid sturgeon (*Huso dauricus* × *Acipenser schrenckii*) is an economically important species in China. With the increasing aquaculture of hybrid sturgeon, the bacterial diseases are a great concern of the industry. In this study, *de novo* sequencing was used to compare the difference in transcriptome in spleen of the infected and mock infected sturgeon with *Aeromonas hydrophila*. Among 187,244 unigenes obtained, 87,887 unigenes were annotated and 1,147 unigenes were associated with immune responses genes. Comparative expression analysis indicated that 2,723 differently expressed genes between the infected and mock-infected group were identified, including 1,420 up-regulated and 1,303 down-regulated genes. 283 differently expressed anti-bacterial immune related genes were scrutinized, including 168 up-regulated and 115 down-regulated genes. Ten of the differently expressed genes were further validated by qRT-PCR. In this study, toll like receptors (TLRs) pathway, NF-kappa B pathway, class A scavenger receptor pathway, phagocytosis pathway, mannose receptor pathway and complement pathway were shown to be up-regulated in *Aeromonas hydrophila* infected hybrid sturgeon. Additionally, 65,040 potential SSRs and 2,133,505 candidate SNPs were identified from the hybrid sturgeon spleen transcriptome. This study could provide an insight of host immune genes associated with bacterial infection in hybrid sturgeon.

## Introduction

Sturgeon is an important fish species farmed worldwide, which has significant economic value as an animal protein source, including caviar and meat^1,2^. It was estimated that in 2014 more than 85% of global sturgeon production came from China, and ranked as the first producer country in the world^2^. *Acipenser schrenckii* and *Huso dauricus* are cultured as pure species and interbred to produce commercially valuable and fertile hybrids^2^.The hybrid sturgeon (*Huso dauricus* × *Acipenser schrenckii*) is one of the five dominant sturgeon strains that was widely bred in China for caviar and meat production^3^, showing the advantage on growth and disease resistant^4,5^. However, with the rapid development of aquaculture, outbreaks of disease caused by bacterial infection lead to high mortality and catastrophic economic losses in hybrid sturgeon aquacultured^6^. The most severe bacterial disease in farmed sturgeons is *Aeromonas hydrophila* infection in China^7^. *Aeromonas hydrophila* is the major pathogen in cultured sturgeon species, including hybrid sturgeon, Amur sturgeon, Siberian sturgeon and Russian sturgeon^8–12^, and the mortality even reached 100%^7^. In the previous study, outbreaks of *Aeromonas hydrophila* infectious diseases have been identified in several provinces in the middle, western and eastern parts of China and we have isolated eight pathogenic *Aeromonas hydrophila* strains^3,13^. *Aeromonas hydrophila* infection caused septicemia with muscle ulcer, gastroenteritic hemorrhage, ascites and cloacal hemorrhaging^8,9,12,13^. As an important aquaculture species in recent years, it is important to understand the defense mechanism of immune system for the control of bacterial infection in hybrid sturgeon (*Huso dauricus* × *Acipenser schrenckii*) aquaculture. Crossbreeding has been used to improve performances of fish growth and disease resistance^14^. The hybrid catfish (female channel catfish × male blue catfish) showed higher postchallenge antibody level and survival ratio than the blue catfish, demonstrating the greater resistance to ESC (enteric septicemia of catfish)^14^. The immunized hybrid catfish developed high antibody titers, and showed high survival after parasitic infection^15^.

To date, genomic information of sturgeon species is extremely scarce, which limits the study of host-pathogen interaction. Deep RNA-sequencing technologies allow transcriptomic analysis of genome reference-free species and non-model organisms at a higher resolution^16,17^. Transcriptome analysis using Illumina sequencing technology has been reported in pathogenic mechanisms of many non-model organisms, such as Chinese sturgeon^18^, Amur sturgeon^19^, Chinese giant salamander (*Andrias davidianus*)^20^, mandarin fish (*Siniperca chuatsi*)^17^, mitten crab (*Eriocheir sinensis*)^16^ and Japanese sea bass (*Lateolabrax japonicus*)^21^. Although the immune-related genes of Chinese sturgeon have been characterized previously, the activity of immune pathway during bacterial infection is still unknown.

This study represents the first report of transcriptome analysis of hybrid sturgeon infected with *Aeromonas hydrophila*. The comparative analysis of transcriptome data provides an insight into the anti-bacterial immunity of hybrid sturgeon. The different genes expression and enrichment analysis of pathways may contribute significant new information regarding the pathogenic mechanisms of the bacterial and the host anti-bacterial immune mechanisms.

## Results

### Transcriptome sequencing and assembly

137,779,578 and 138,474,832 raw reads from the spleen of *Aeromonas hydrophila* infected and mock infected hybrid sturgeons, respectively, were obtained using Illumina Hiseq. 2000 (paired reads, 90 bp) deep sequencing analysis. The data was refined by discarding low-quality reads that contained unknown bases or whose length was lower than 20 nucleotides after removal of the adaptors and low-quality bases. After filtering, the infected and mock infected library generated 135,705,954 cleaned reads and 136,407,246 cleaned reads, respectively. The total length of these reads was 12.21 × 10^9^ and 12.28 × 10^9^ base pairs for infected and mock infected samples, respectively. The Q20 percentage (the percentage of sequences with a sequencing error rate lower than 1%) was over 97%, and GC percentage was over 47% for both samples (Table 1). All high-quality reads were deposited in the National Central for Biotechnology Information (NCBI) Sequence Read Archive (SRA) and can be accessed under the accession number (SRR4067580).

**Table 1:** Summary of sequencing read results.

	Bacteria infected	Mock infected
Total raw reads	137,779,578	138,474,832
Total clean reads	135,705,954	136,407,246
Total clean nucleotides(bp)	12,213,535,860	12,276,652,140
Q20 percentage	98.11%	97.95%
GC percentage	47.81%	47.80%

**Figure 1:**
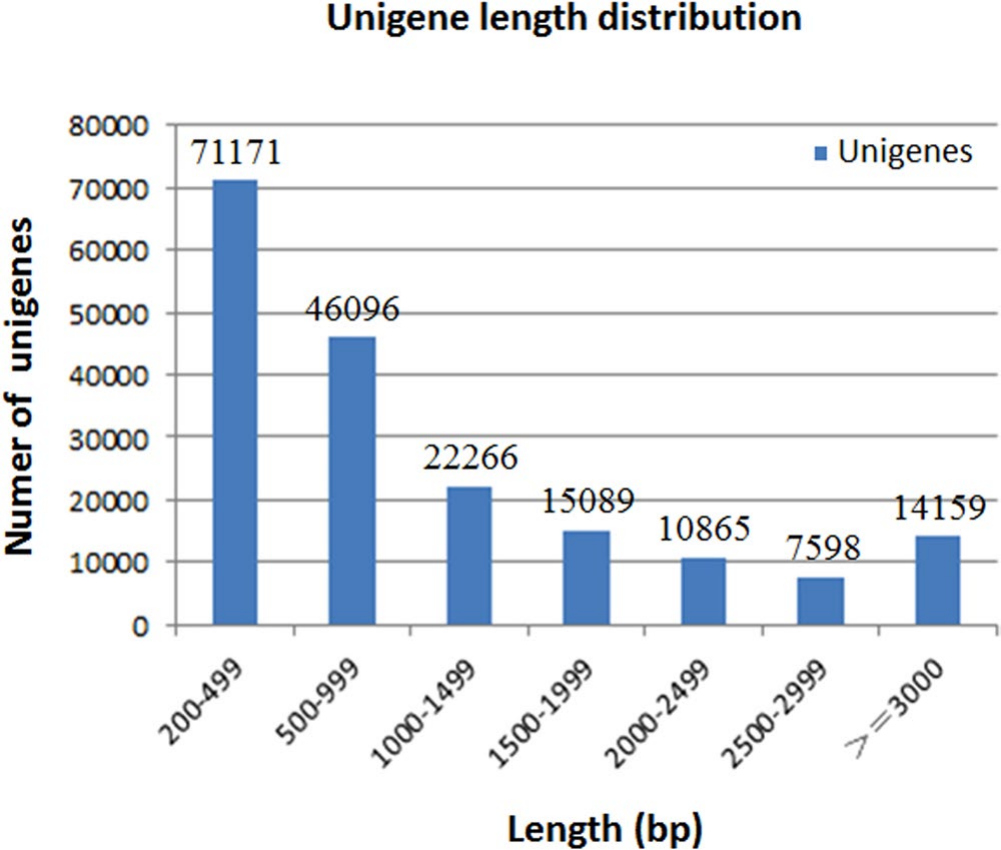
Length distribution of assembled unigenes. The X axis represents unigene size and the Y axis represents the number of unigenes.

A summary of all contigs and unigenes assembly was presented in Table 2. The total length and number of contig were 96,088,427 bp and 308,709, respectively, and the average contig length was 311 bp (N50 = 518). The total length and number of unigene were 197,787,478 bp and 187,244, respectively, and the average unigene length was 1056 bp (N50 = 1934). 117,267 (62.63%) of the unigenes were clustered in a group with 200–1000 bp in length, and 14,159 (7.56%) of the unigenes were longer than 3000 bp. The length distribution of these unigenes was shown in Fig. 1. The unigenes were deposited in the NCBI Transcriptome Shotgun Assembly (TSA) and can be accessed by the accession number (GGQL00000000).

**Table 2:** Summary of assembly results.

	Total length (bp)	Number	Average length (bp)	N50
Contig	96,088,427	308,709	311	518
Unigene	197,787,478	187,244	1056	1934

### Function annotation and classification

The unigenes were blasted against Nr, Swiss-Prot, COG and KEGG databases using BLASTx with a cut-off E value of 10^−5^, as well as against Nt database using BLASTn with a cut-off E value of 10^−5^. 87.887 (46.9%) unigenes were annotated in at least one of the databases. Among them, 70,197 (79.9%), 73,903 (84.1%), 66,713 (75.9%), 26,506 (30.2%) and 55.285 (62.9%) unigenes were found in Nt, Nr, Swiss-Prot, COG and KEGG databases (Table 3), respectively. Approximately, 77,556 (41.4%) unigenes have reliable coding sequences (CDS) by BLASTx and ESTscan analysis^22,23^. 74,061 (39.6%) of the unigenes had good comparability with known gene sequences in existing species based on analysis with the Nr, Swiss-Prot, KEGG and COG databases for functional annotation.

**Table 3:** Annotation of unigenes of transcriptomic profiles.

Database	Number of annotated unigenes	Percentage of annotated unigenes
Nr	73.903	84.1%
Nt	70,197	79.9%
Swiss-Prot	66,713	75.9%
KEGG	55,285	62.9%
COG	26,506	30.2%
GO	22,303	25.4%
Total	87,887	100%

**Figure 2:**
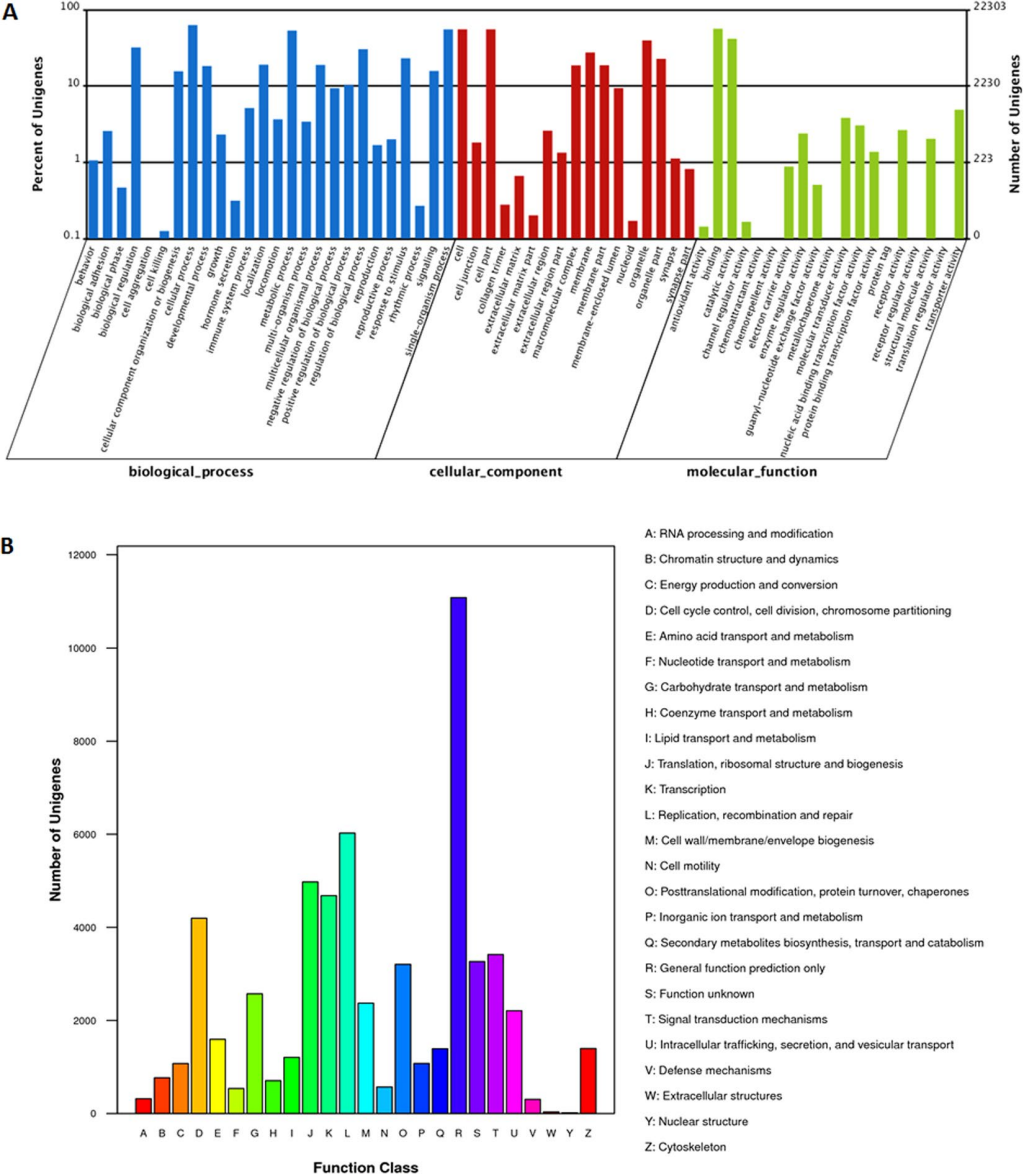
GO function annotation and COG function classification. (**A**) GO function annotation. All 22,303 unigenes were aligned to the GO database, and were grouped into three major functional categories and 62 sub-categories. Right Y axis written in roman represents the number of unigenes in a category. Left Y axis written in roman represents the percentage of a specific category of unigenes in each main category. The X axis is GO sub-categories. (**B**) COG classification of putative proteins. All 26,506 putative proteins were aligned to the COG database and were classified into 26 molecular families. The Y axis written in roman represents the number of unigenes in a specific functional cluster. The X axis is COG families.

Based on Nr annotations, the Gene Ontology (GO) classification system was used to classify the possible functions of the unigenes. A total of 22,303 (25.4%) unigenes were successfully assigned to three major functional categories (biological process, cellular component and molecular function) and 62 sub-categories (Fig. 2A). For the biological process, the top six largest categories were “cellular process” (14,035), “single-organism process” (12,314), “metabolic process” (11,899), “biological regulation” (7,148), “regulation of biological process” (6,771) and “response to stimulus” (5,147). And 1,147 unigenes belonged to immune system. For the cellular component category, the top three largest categories were “cell” (12,329), “cell part” (12,329) and “organelle” (8,834). For the molecular function category, 12,547 and 9,284 unigenes were classified into the sub-categories “bingding” and “catalytic activity” respectively.

To classify orthologous gene products, 26,506 (30.2%) unigenes were classified functionally into 26 COG classifications (Fig. 2B). The largest category was “general function prediction only” (11,077 unigenes, 41.8%), followed by “replication, recombination and repair” (6,026 unigenes, 22.7%), “translation, ribosomal structure and biogenesis” (4,975 unigenes, 18.8%), “transcription” (4,681 unigenes, 17.7%) and “cell cycle control, cell division, chromosome partitioning” (4,195 unigenes, 15.8%). Additionally, 3,265 (12.3%) unigenes were annotated as “function unknown”.

**Figure 3:**
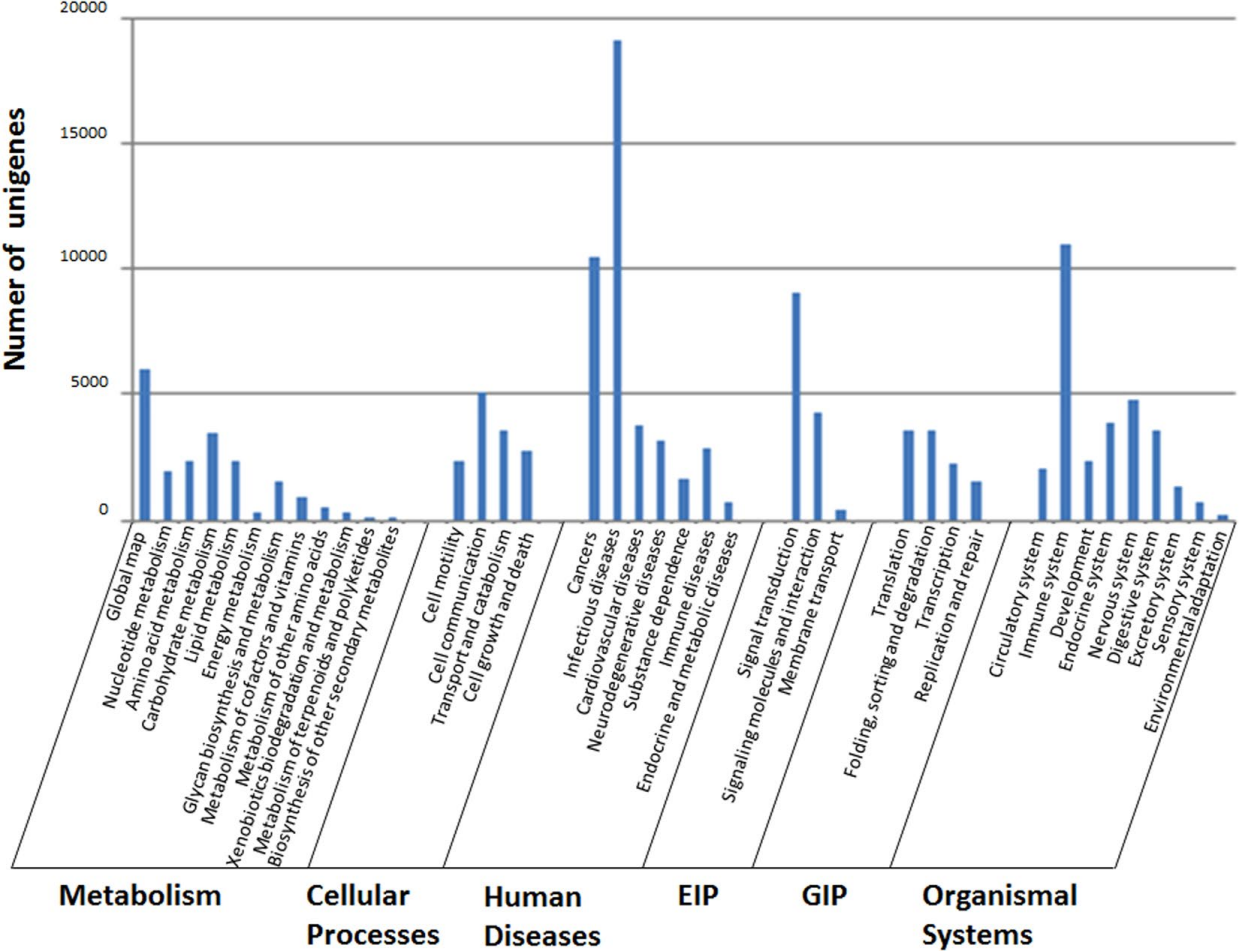
KEGG categories of unigenes. All unigenes were annotated using KEGG Automatic Annotation Server for pathway information. The categories GIP and EIP stand for genetic information processing and environmental information processing, respectively. The X axis is KEGG pathway category and Y-axis written in roman indicates the number of unigenes in each category respectively.

Furthermore, the KEGG database was used to analyze the annotation and data of metabolic pathways. Among the 87,887 annotated unigenes, 55,258 were grouped into six categories comprised of 259 known KEGG pathways (Fig. 3). 20,099 (36.37%) KEGG-annotated unigenes were assigned to metabolic pathways, 19,049 (34.47%) to infectious disease, 10,958 (19.88%) to immune system, 10,475 (18.96%) to cancer pathway, 9,102 (16.47%) to signal transduction.

### Differential expression analysis

**Figure 4:**
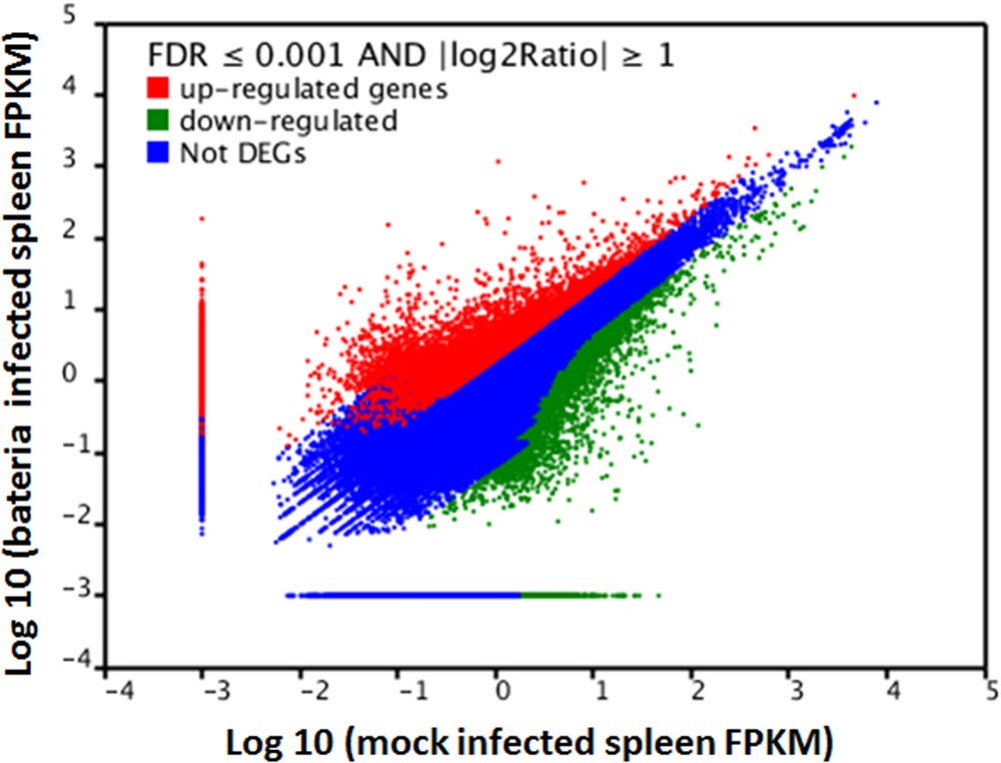
Identification of differentially expressed genes between bacteria infected and mock infected groups. “Not DEGs” indicate “not differentially expressed genes”. The X axis contains Log10 of transcript per million of the mock infected group and the Y axis indicates Log10 of transcript per million of the bacteria infected group. Limitations are based on P ≤ 0.01, and the absolute value of Log_2_ is greater than 1.

To identify gene expression changes between bacteria infected and mock infected samples, FPKM method was used to calculate the expression levels of genes. 2,723 genes were found to be expressed differently between bacteria infected and mock infected groups. 1,420 genes were up-regulated and 1,303 genes were down-regulated with an FDR ≤0.001 and ratios larger than 2. The up-regulated genes (red spots), down-regulated genes (green spots) and no differential expression genes (blue spots) distribution trends were shown in Fig. 4. Among these differently expressed genes, 1,233 genes were assigned to at least one of the Nr, Nt, Swiss-Prot, KEGG and COG databases, in which 700 genes were up-regulated and 533 genes were down-regulated.

**Figure 5:**
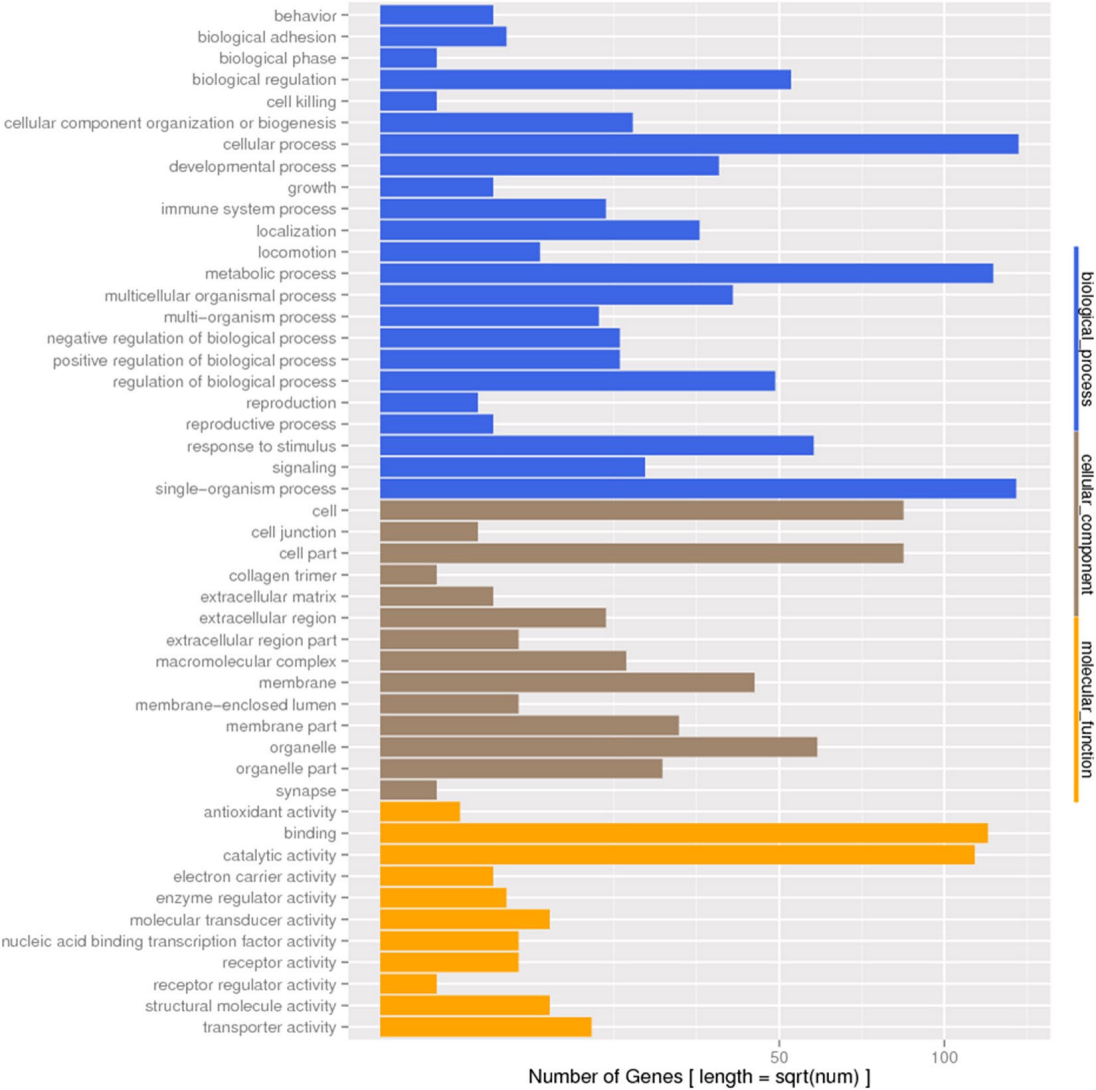
GO functional classification of the differentially expressed genes between bacteria infected and mock infected groups. The Y axis written in roman represents the number of unigenes in each category. The X axis is GO categories.

GO enrichment analysis was conducted to clarify the biological functions of all differentially expressed unigenes (DEGs) identified. All DEGs were mapped to each term of the GO database, and the GO terms with a corrected P value ≤ 0.05 were defined as significantly enriched in DEGs. The results indicated that all 2,723 DGEs were enriched in three categories (Fig. 5): biological process (23 sub-categories), cellular component (13 sub-categories) and molecular function (11 sub-categories). In biological process category, there were 15 sub-categories showed significant enrichment, including “cellular process”, “single-organism process”, “metabolic process”, “response to stimulus”, “biological regulation”, “regulation of biological process”, “multicellular organismal process”, “development process”, “localization”, “signaling”, “cellular component organization or biogenesis”, “negative regulation of biological process”, “positive regulation of biological process”, “immune system process” and “multi-organism process”. In cellular component category, there were 8 sub-categories showed significant enrichment, including “cell”, “cell part”, “organelle”, “membrane”, “membrane part”, “organelle part”, “macromolecular complex” and “extracellular region”. In molecular function category, the enriched sub-categories were “binding”, “catalytic activity” and “transporter activity”.

An enrichment analysis of the KEGG pathways was also performed to clarify the biological pathways of all DEGs. These DEGs were significantly enriched in 12 pathways including “metabolic pathways” (90 DGEs, 14.06%), “phagosome” (57 DGEs, 8.91%), “amoebiasis” (55 DGEs, 8.59%), “tuberculosis” (50 DGEs, 7.81%), “rheumatoid arthritis” (44 DGEs, 6.88%), “influenza A” (42 DGEs, 6.56%), “transcriptional misregulation in cancer” (42 DGEs, 6.56), “cytokine-cytokine receptor interaction” (42 DGEs, 6.56%), “hematopoietic cell lineage” (42 DGEs, 6.56%), “NF-kappa B signaling pathway” (40 DGEs, 6.25%), “Epstein-Barr virus infection” (40 DGEs, 6.25%) and “Straphylococcus aureus infection” (39 DGEs, 6.09%) (see Supplementary Table S1 online). The mostly enriched pathway associated with bacteria defense was “phagosome”, with 27 DGEs up-regulated and 30 DGEs down-regulated (Table 4), which may be involved in bacteria degradation, pathogens killing and the regulated processing of their proteins for antigen presentation^24^.

**Table 4:** The gene cluster in phagosome pathway.

Gene ID	log2 Ratio (bacteria infected spleen/ mock infected spleen)	Annotation
K05692: CL12904.Contig1_All	2.3	F-actin
K06751: Unigene352_All	15.9	MHC I
Unigene83245_All	10.4	MHC I
Unigene88060_All	4.1	MHC I
Unigene87158_All	3.8	MHC I
K06752: Unigene83861_All	6.1	MHC II
K06498: CL2313.Contig1_All	2.4	FcyR
K01330: Unigene91192_All	11.8	CR1
Unigene59124_All	5.9	CR1
K10159: CL1556.Contig2_All	3.0	TLR2
CL22561.Contig1_All	2.3	TLR2
K06560: CL5583.Contig1_All	3.4	MR
CL5583.Contig3_All	2.7	MR
Unigene61749_All	2.7	MR
K06563: CL25767.Contig4_All	3.4	DCSIGN
K13884: CL41.Contig1_All	3.4	MARCO
CL41.Contig7_All	3.4	MARCO
CL41.Contig16_All	3.2	MARCO
CL41.Contig4_All	3.2	MARCO
CL41.Contig5_All	3.2	MARCO
CL41.Contig10_All	3.1	MARCO
CL41.Contig14_All	2.9	MARCO
CL41.Contig15_All	2.5	MARCO
K07374: Unigene87966_All	10.2	TUBA
K13240: CL23117.Contig1_All	3.1	NOS
CL23117.Contig3_All	3.1	NOS
K08008: Unigene10283_All	2.1	gp91
K06751: Unigene6484_All	−2.5	MHC I
Unigene6768_All	−2.2	MHC I
Unigene6578_All	−2.2	MHC I
CL6799.Contig6_All	−2.0	MHC I
K06752: CL458.Contig5_All	−3.0	MHC II
Unigene7108_All	−2.7	MHC II
CL458.Contig6_All	−2.6	MHC II
Unigene7236_All	−2.0	MHC II
K01330: CL3648.Contig2_All	−4.9	CR1
CL17607.Contig2_All	−3.9	CR1
K06856: CL7722.Contig1_All	−6.1	IG
CL6920.Contig1_All	−4.8	IG
CL6920.Contig5_All	−4.5	IG
CL17055.Contig1_All	−4.3	IG
CL6920.Contig2_All	−3.9	IG
CL8987.Contig3_All	−2.9	IG
CL6920.Contig3_All	−2.8	IG
CL8987.Contig4_All	−2.8	IG
Unigene74025_All	−2.4	IG
CL8222.Contig4_All	−2.3	IG
CL8987.Contig2_All	−2.1	IG
CL8987.Contig1_All	−2.1	IG
CL8222.Contig6_All	−2.1	IG
CL17055.Contig2_All	−2.1	IG
K10062: CL6867.Contig3_All	−2.6	collectins
K06563: CL6867.Contig3_All	−2.6	DCSIGN
K07375: Unigene21003_All	−5.8	TUBB
Unigene22807_All	−2.8	TUBB
K00921: CL21876.Contig2_All	−5.1	PIKFYVE
K01365: Unigene49001_All	−2.1	cathepsin
K01368: Unigene59541_All	−2.0	cathepsin

### Differential expression immune-relevant genes analysis

In this study, 283 annotated genes, including 168 up-regulated genes and 115 down-regulated genes, were found to be involving in various anti-bacterial immune-relevant pathways, they were further grouped into 6 sub-categories as follows: pattern recognition genes, complement system, inflammatory cytokines and receptors, T-cell and B-cell antigen activation, antigen presenting and regulators, adapters, effectors and signal transducers, other genes related to immune cell response (Table 5).

**Table 5:** Differentially expressed genes (DEGs) associated with immune response between bacteria infected and mock infected hybrid sturgeon.

Catalogs	Consensus number
***Pattern recognition genes***	
Toll like receptor (2,5,8)	7
Scavenger receptor	12
Mannose receptor	3
C-type lectin	4
**NACHT, LRR and PYD domains-containing**	
-protein (NALP)	1
LPS-binding/anchor protein	2
***Complement System***	
C1q	1
Complement factor (B, D)	6
Complement receptor	1
Complement component	3
***Inflammatory Cytokines and Receptors***	
IL (1,6, 8,11,12) and relevant	20
IL receptor and relevant	5
IFN-induced proteins and relevant	13
TITIN	6
Chemokine	4
Chemokine receptor	3
Matrix metalloproteinase	4
Angiopoietin and relevant	3
Family with sequence similarity (FAM)	4
P2X purinoceptor	1
Pentraxin-related protein	1
Prostaglandin synthase	3
Hyaluronan synthase	1
Myelomonocytic growth factor	1
Disintegrin and metalloproteinase	1
Chitinase 1 TRAF3IP2	1
***T-cell and B-cell Antigen Activation***	
TCR	2
Immunoglobulin and relevant CD	40
B cell linker protein (BLNK)	1
B2m/b2 gene	5
Homeobox protein	5
RAG1	1
CBLB	1
GTPase IMAP family (4,7,8)	9
Pre-B lymphocyte protein	1
Purine nucleoside phosphorylase	1
**Carcinoembryonic antigen-related cell adhesion**	
-molecule (CEACAM1)	1
Fc-receptor	1
VLRA	1
**Receptor-type tyrosine-protein phosphatase eta**	
(R-PTP-ETA)	1
***Antigen Processing and Regulators***	
MHC I/II	23
Integrin α/β	3
TNF / TNR	12
proteasome	1
Mixed lineage kinase domain (MLKL)	1
Minor histocompatibility antigen H13 (HM13)	1
***Adapters, Effectors and Signal Transducers***	
TRAF	4
Calmodulin	1
NF κB	1
***Other Genes Related to Immune Cell Response***	
TRAF associated NF-κB activator -binding kinase	1
CD (84,209,276)	4
Apolipoprotein	5
Activator protein 1 (AP-1)	1
Tripartite motif-containing protein (TRIM)	2
NF κB inhibitor	1
Antimicrobial peptide	7
Serotransferrin1	1
Ferritin	2
Hepcidin1	1
Hsp70	2
Macroglobulin	2
Microtubule-associated	1
Nitric oxide synthase	5
Deleted in malignant brain tumors (DMBT)	2
Protein S100-A	1
Cytochrome b-245 heavy chain (CYBB)	1
Caspase relevant	3
Apoptotic relevant	4
Immune-responsive gene (IRG)	2
Protein AF1q	1
Fos relevant	1
Transcription factor MafB	1
L-amino-acid oxidase (LAO)	2
Cathepsin	1
Dendritic cell relevant	1

### *SSR* and *SNP* discovery

**Figure 6:**
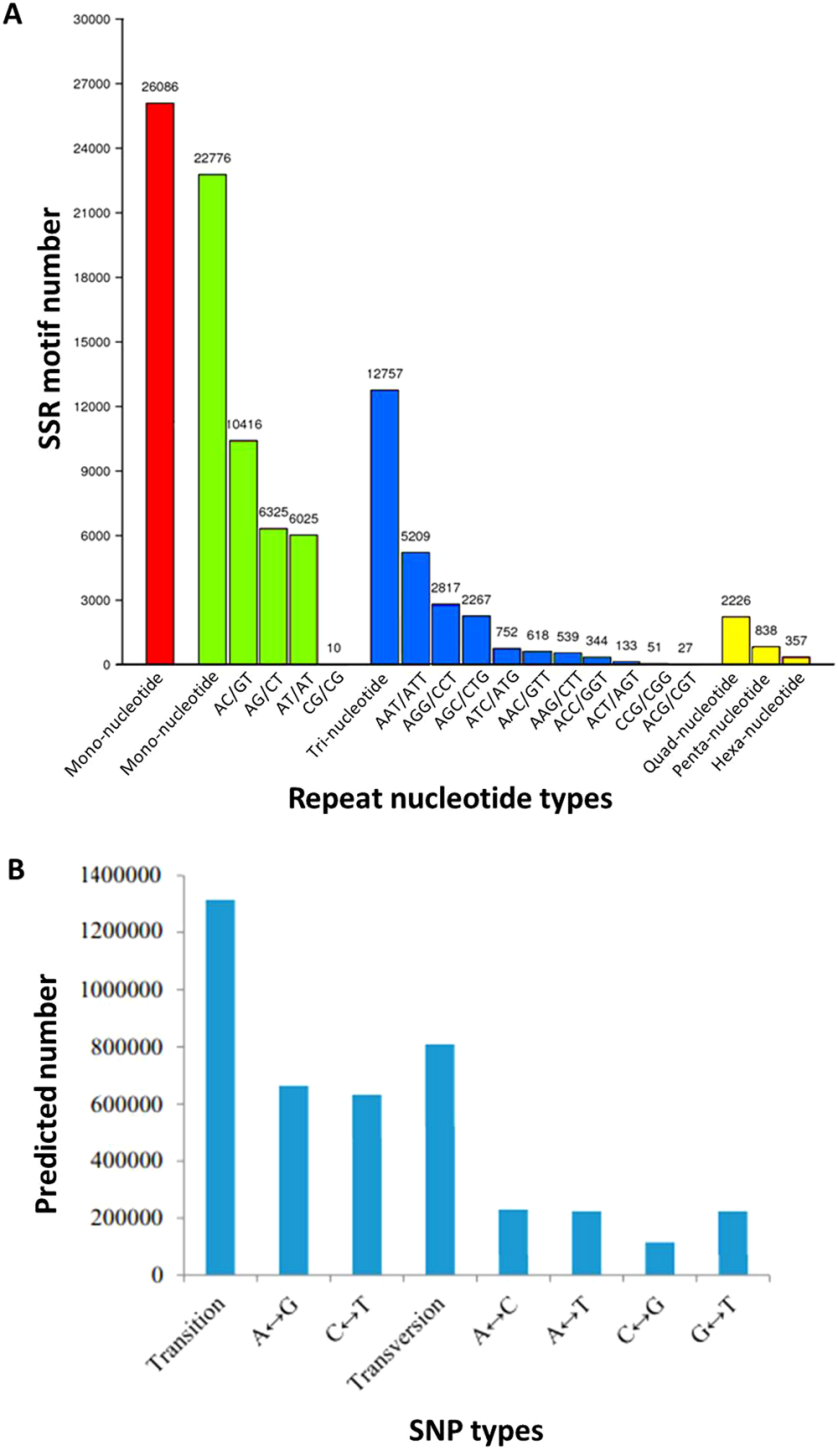
Distribution of SSRs and SNPs in the spleen transcriptome. (**A**) Distribution of SSRs among different nucleotide types, a total of 65,040 SSRs were identified. (**B**) Distribution of SNPs based on different types, a total of 2,133,505 putative SNPs were identified.

Simple sequence repeats (SSRs) is an efficient tool for performing quantitative trait loci (QTL) analysis and constructing genetic linkage(s) due to their high diversity and abundance. Using the MicroSAtellite (MISA), 65,040 potential SSRs from 187,244 unigenes were identified from the hybrid sturgeon. Among them, the most frequent repeat motifs were mononucleotide SSR motifs (26,086, 40.10%), followed by dinucleotide (22,776, 35.2%), trinucleotide (12,757, 19.61%), quadnucleotide (2,226, 3.42%), pentanucleotide (838, 1.29%) and hexanucleotide (357, 0.55%) (Fig. 6A). 210 motif sequence types were identified, with which mono-, di-, tri-, quad-, penta- and hexa-nucleotide repeats were 2, 4, 10, 28, 69, 97 types, respectively. Of the dinucleotide, AC/GT class was the most common and accounted for 45.73%. Among the trinucleotide repeats, AAT/ATT, AGG/CCT and AGC/CTG were the three predominant types and accounted for 40.83%, 22.08% and 17.78%, respectively.

Compared with the assembled transcript sequences using Samtools and VarScan, 2,133,505 candidate single nucleotide polymorphisms (SNPs) were identified. Of these SNP candidates, 1,330,390 SNPs were putative transitions (Ts), and 803,115 SNPs were putative transversions (Tv) with a mean Ts: Tv ratio of 1.66. The SNPs were then categorized into 4 classes, including class 1 (C/A, A/C, T/G and G/T) at 437,968 (20.53%), class 2 (C/T, G/A, T/C and A/G) at 1,330,390 (62.36%), class 3 (C/G and G/C) at 149,199 (6.99%), and class 4 (A/T and T/A) at 215,948 (10.12%) (Fig. 6B).

### Experimental validation

**Figure 7:**
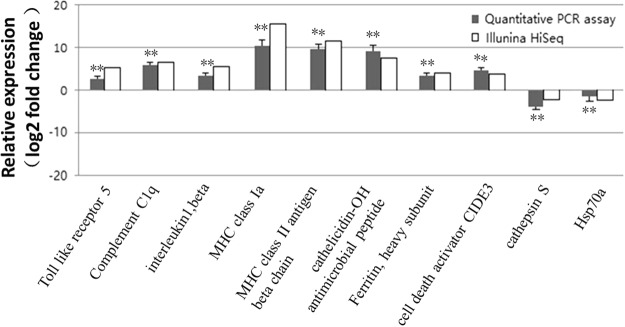
Comparison of log2 expressions of ten differentially expressed genes determined by Illumina HiSeq. 2000 sequencing and qRT-PCR. These DEGs were amplified in sturgeons spleens 7 h post bacterial infection. β-actin was used as an internal reference gene. Positive and negative log2 expression ratios represent up- and down- regulation after bacterial infection respectively. The asterisk indicate significant different (**P ≤ 0.05) from the control.

To validate the integrity of RNA-seq results, selected unigenes (Table 6) with complete coding regions were evaluated by reverse transcription quantitative real-time PCR (q-PCR) (Fig. 7). The log2 fold change of each gene values obtained from RNA-seq and q-PCR were as follows: Toll like receptor 5 (TLR5) (4.73 VS 2.34), complement C1q (C1) (6.11 VS 5.67), interleukin 1β (IL-1β) (4.81 VS 3.39), major histocompatibility complex class Ia chain (MHC Ia) (15.94 VS 10.24), MHC class II antigen β chain (MHC IIβ) (11.16 VS 9.59), cathelicidin-OH antimicrobial peptide (CAMP) (7.71 VS 9.00), ferritin (3.32 VS 2.90), cell death activator CIDE-3 (CIDE-3) (3.02 VS 4.60), cathepsin S (−2.0 VS −4.26) and heat shock protein 70a (Hsp70a) (−2.79 VS −1.64.). The internal control was β-actin, and the log2 fold change obtained from q-PCR was measured with the 2^−ΔΔCT^ method and normalized by the median expression of β-actin. Even though the folds of changes were not exactly the same, however they showed the identical up-regulated or down-regulated patterns of these ten genes in both assays. This demonstrated the reliability of RNA-seq results and indicates the necessity for further identification of immune relevant genes in sturgeon.

**Table 6:** Primers used for qRT-PCR verification of differentially expressed genes.

Genen name	Forward primer (5′-3′)	Revers primer (5′-3′)
Toll like receptor 5	GCGATGGCTCGGAAGAAGTT	CCAACAGCAGTGTCTGCCCT
Complement C1q	GTGCTTTCCCACCATCCAGT	GCTCAAGACGCTGACCAAAA
interleukin1,beta	TGATGCTGGAGGTGAATCCC	CCGAGTCGCTTATCGAGTGG
MHC class Ia	CCCTCAGACTTTGCCATCCA	CCCTGAGTTTGTGACGGTGG
MHC class II antigen	GACAACAGGTGGTCCAGTGG	TCTGCCCATGCTGTACTGTG
**Beta chain**		
cathelicidin-OH	GGAATCCTCAGCTTTTGCCA	TCGTCCCCTACTTCCATTGC
**Antimicrobial peptide**		
Ferritin, heavy subunit	GTTGATGGCTGCCTCGCAGT	CATCATCGCCGCTTCACTCA
cell death activator CIDE3	ATCTTCTTGGCCCCGTAGCA	CAAGTCGAACCCCCGTGATT
cathepsinS	GCTCTGTTGCTCTCGTCT	CTGACGAAGATGACAAGC
Hsp70a	ATGCAGGGGACAGCACAGCT	TTGACTCGAACCCTCCCCGC
β-actin	GCAGGAAGATCCAGCAAAAG	GCTTCCTCTTGCTCCATCTG

## Discussion

The transcriptome represents the complete repertoire of RNA transcripts in a cell investigated. Recently, many reports revealed that the transcriptome analysis is a great tool in deciphering the functional complexity of the genome and in obtaining a better understanding of cellular activities in organisms, including growth, development, disease, and immune defense^21,25^. In this study, the transcriptome profiles of the spleen from *Aeromonas hydrophila* infected and mock infected hybrid sturgeons (*Huso dauricus* × *Acipenser schrenckii*) were analyzed and compared. A total of 187,244 unigenes were obtained with a mean length of 1056 bp. The length distribution showed that 62.63% of the unigenes were clustered in a group with 200–1000 bp in length and 7.56% of the unigenes were longer than 3000 bp, which was similar to previous transcriptomes of the Amur sturgeon spleen and liver that were based on Illumina sequencing^19,26^. Hence, the transcriptomes of the hybrid sturgeon spleens were effectively established and could be utilized for further analysis. There were 2,723 differential expressed genes between the two groups, including 1,420 up-regulated and 1,303 down-regulated genes. The spleen is an important immune organ in fishes, the previous comparative transcriptome of Amur sturgeon spleen identified 125 DEGs between *Yersinia ruckeri* infection and mock-infection groups and these DEGs could be divided into 16 immune-related KEGG pathways^26^. Therefore, we focused on genes related to immune responses after *Aeromonas hydrophila* infected. 283 anti-bacterial immune related genes were found to be differently expressed between the two groups were scrutinized and discussed, including 168 up-regulated and 115 down-regulated genes.

### Pattern recognition genes

**Figure 8:**
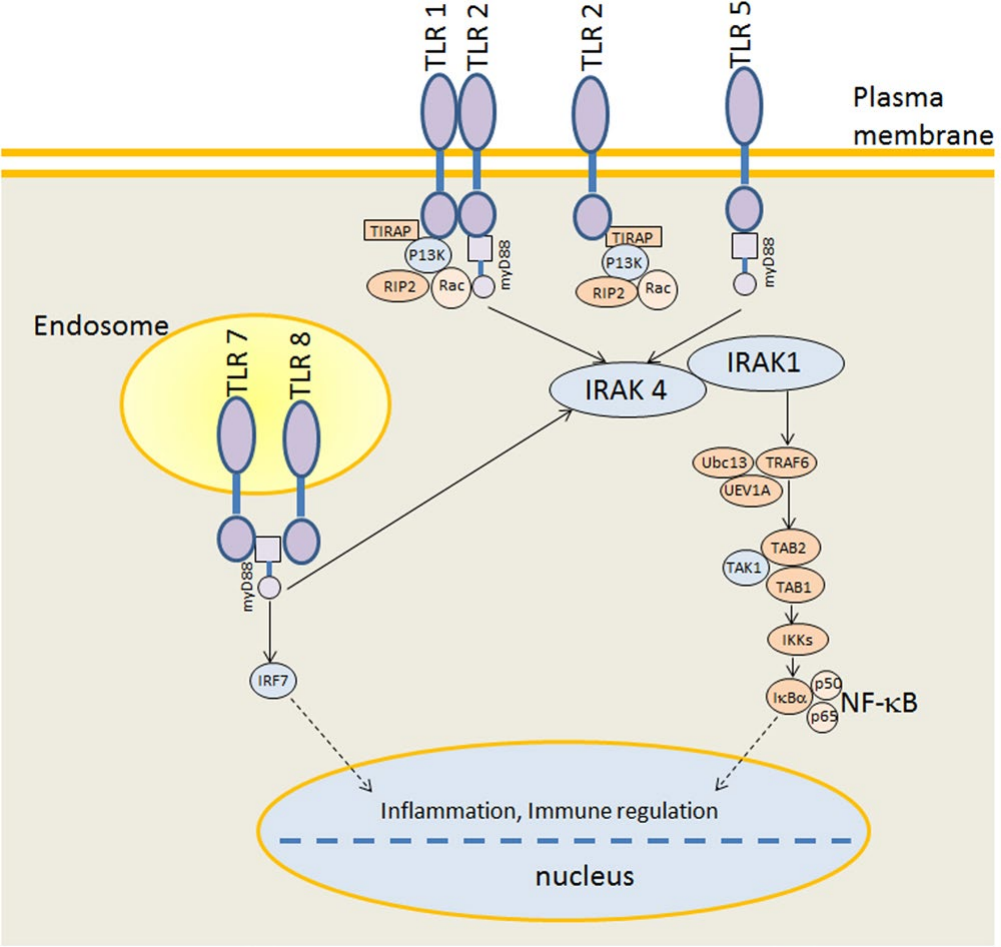
Proposed TLR 2, 5, 8 signaling pathway. The proposed TLR signal pathway of sturgeon was constructed based on knowledge of TLR signaling in teleost fish. However, most interactions have to be confirmed experimentally.

The pattern recognition receptors (PRRs) could recognize specific surface components of microorganisms^27^. The Toll like receptor (TLR) is one of the most important classes of PRRs that play crucial roles in initiating inflammatory responses and shaping adaptive immunity^28^. In mammals, the human genome contains 10 functional TLRs whereas the mouse genome contains 12 TLRs, and many more TLR genes are identified in several fish species (TRL18, 19, 20, 22, 25, 26) than in mammalian species due to the presence of duplicated TLRs and fish-specific TLRs^18,29–32^. In mammalian, TRL 1, 2, 4, 5, 6 and 10 are located on the cell membrane, while TRL 3, 7, 8 and 9 functions within the cytoplasm^33^. Activation of TLRs stimulates NF-kappa B pathway through MyD88-dependent pathway and finally induces pro-inflammatory cytokine (IL, tumor necrosis factor TNF)^33^. In this report, TRL 1–9, 13 and 22 were detected by transcriptome analysis, they belong to six TLR families (TLR1, TLR3, TLR4, TLR5, TLR7, and TLR11) found in vertebrate taxa. TRL 1–9 and 13 are normally found in mammals, while TLR22 is “fish-specific” TLR. Among them, TRL 2, 5 and 8 were up-regulated significantly in the spleen of Aero*monas hydrophila* infected hybrid sturgeons, suggesting that the TLR pathway is activated in the infected fish. TRL5 was significantly up-regulated in *Yersinia ruckeri* -infected Amur sturgeon through transcriptome analysis^19^. Fish have both a membrane and a soluble form of TLR5 that senses bacterial flagellin. TLR5 is involved in recognizing bacterial flagellin and after binding, it triggers myeloid differentiation primary response gene 88 (MyD88)-dependent signaling pathway to induce pro-inflammatory cytokines^34^. It is noteworthy that *Aeromonas hydrophila* possesses flagellin, we speculated that TLR5 was up-regulated by triggering of flagellin in this study. Interestingly, in the present study, the majority of the DEGs associated with “NF-kappa B signaling pathway” were strongly induced in infected sturgeon. There are 13 genes up-regulated (IL-1β, IL8, TNF receptor 1, interleukin-1 receptor-associated kinase 1 (IRAK 1), TNF receptor associated factor (TRAF) 1/2, TRAF 2/5, TRAF 2/6, TRAF 2, TRAF 3, epidermal growth factor receptor kinase (Btk), prostaglandin H synthase (COX2), vascular cell adhesion protein (VCAM) 1, C-C motif chemokine 19), 4 genes down-regulated (TCR, BCR, IRAK4, IL8) and eighteen IL (1β, 6, 8, 11, 12β) genes up-regulated in the infected fish (Table 7). These results suggested that TLRs activate NF-kappa B pathway induced inflammatory response to defense against *Aeromonas hydrophila* infection in hybrid sturgeon. The TLR mechanisms are conserved from fish to mammals throughout vertebrate evolution. A putative draft of TLR signaling pathways in hybrid sturgeon based on the knowledge of TLR signaling in teleost fish was constructed (Fig. 8).

**Table 7:** Representatives of DEGs associated with immune response between bacteria infected and mock infected hybrid sturgeon.

Catalogs or gene ID	Homologous function	log2 (fold change)	Fold Change
***Pattern recognition genes***			
CL1556.Contig2_All	Toll like receptor 2	2.96	7.78
Unigene11032_All	Toll like receptor 5	4.73	26.54
CL27267.Contig5_All	Toll like receptor 8	2.88	7.36
CL762.Contig5_All	scavenger receptor cysteine-rich-protein	3.02	8.11
CL762.Contig7_All	scavenger receptor cysteine-rich-protein	2.99	7.94
CL762.Contig5_All	scavenger receptor cysteine-rich-protein	2.71	6.54
CL41.Contig1_All	macrophage receptor MARCO	3.40	10.56
CL41.Contig4_All	macrophage receptor MARCO	3.22	9.32
CL41.Contig5_All	macrophage receptor MARCO	3.23	9.38
CL41.Contig7_All	macrophage receptor MARCO	3.38	10.41
CL41.Contig10_All	macrophage receptor MARCO	3.14	8.82
CL41.Contig14_All	macrophage receptor MARCO	2.94	7.67
CL41.Contig15_All	macrophage receptor MARCO	2.51	5.70
CL41.Contig16_All	macrophage receptor MARCO	3.14	8.82
CL25767.Contig4_All	C-type lectin domain family 4-member D	3.38	10.41
Unigene61749_All	C-type mannose receptor 1	2.66	6.32
CL5583.Contig1_All	C-type mannose receptor 2	3.43	10.78
CL13419.Contig3_All	NACHT, LRR and PYD domains-containing protein 6	−5.27	0.026
***Complement System***			
Unigene11133_All	complement C1q	6.11	69.07
Unigene57610_All	complement factor B	3.68	12.82
CL2836.Contig5_All	complement factor D	3.98	15.78
Unigene91192_All	complement component 1	11.78	3516.68
***Inflammatory Cytokines and Receptors***			
CL23561.Contig2_All	interleukin 1, beta	4.81	28.05
Unigene15983_All	interleukin-6	5.89	59.30
CL12506.Contig2_All	interleukin-8	5.06	33.36
Unigene62770_All	interleukin-11	7.56	188.71
Unigene91444_All	interleukin 12, beta	4.57	23.75
CL5726.Contig2_All	interleukin-1 receptor type 2	7.27	154.34
Unigene83458_All	interferon-induced very large-GTPase 1	7.59	192.67
CL10938.Contig3_All	c-C motif chemokine 4	4.17	18.00
CL7831.Contig1_All	c-C motif chemokine 19	2.08	4.23
CL315.Contig1_All	chemokine XC receptor 1	−3.11	0.12
Unigene17815_All	matrix metalloproteinase-13	4.95	30.91
Unigene92305_Al	P2X purinoceptor	10.28	1243.34
Unigene53273_All	prostaglandin E synthase	3.97	15.67
CL7038.Contig4_All	disintegrin and metalloproteinase-domain-containing protein 8	2.2	4.59
Unigene35086_All	TRAF3 interacting protein	4.37	20.68
***T-cell and B-cell Antigen Activation***			
Unigene24755_All	T cell receptor delta chain	−2.20	0.22
CL2256.Contig2_All	T cell receptor gamma chain	−2.35	0.20
CL7722.Contig1_All	immunoglobulin mu heavy chain	−6.11	0.014
CL11233.Contig2_All	immunoglobulin lambda light chain	−4.67	0.039
Unigene64353_All	CD3 epsilon chain	−2.53	0.17
Unigene15817_All	CD4	2.19	0.22
CL17329.Contig1_All	B2m/b2 gene	−2.88	0.14
Unigene44962_All	recombination activating gene 1	−3.39	0.095
CL5381.Contig3_All	E3 ubiquitin-protein ligaseCBLB	2.03	4.08
CL18807.Contig3_All	GTPase IMAP family member 7	−2.23	0.21
CL2313.Contig1_All	Fc-receptor	2.4	5.28
***Antigen Processing and Regulators***			
Unigene352_All	MHC class Ia chain	15.94	62866.33
CL7070.Contig6_All	MHC class II antigen beta chain	11.16	2288.20
CL15301.Contig3_All	integrin α-E	3.94	15.35
CL18887.Contig1_All	integrin β	−7.21	0.0068
CL6743.Contig1_All	tumor necrosis factor receptor-superfamily member 6B	6.38	83.29
CL13406.Contig3_All	proteasome subunit beta	−2.73	0.15
CL1507.Contig6_All	mixed lineage kinase domain	3.72	13.18
Unigene64944_All	minor histocompatibility antigen H13	−2.23	0.21
***Adapters, Effectors and Signal Transducers***			
CL1308.Contig10_All	TNF receptor-associated factor 2	5.91	60.13
CL11338.Contig4_All	calmodulin	2.08	4.23
***Other Genes Related to Immune Cell Response***			
Unigene9686_All	CD276	−2.54	0.17
CL1852.Contig2_All	apolipoprotein L6	−6.22	0.013
Unigene6070_All	AP-1	−2.13	0.23
CL6277.Contig3_All	cathelicidin-OH antimicrobial peptide	7.71	209.38
Unigene5003_All	cathelicidin 2	10.20	1176.27
CL9413.Contig1_All	serotransferrin 1	9.40	675.59
CL3835.Contig6_All	ferritin, heavy subunit	3.32	9.99
CL4584.Contig2_All	hepcidin 1	4.16	17.88
Unigene28855_All	Hsp70a	−2.79	0.14
CL25024.Contig2_All	microtubule-associated protein 1	−2.19	0.22
CL23117.Contig1_All	nitric oxide synthase	3.15	8.88
Unigene12031_All	protein S100-A1	3.57	11.88
Unigene10283_All	cytochrome b-245 heavy chain	2.13	4.38
Unigene31871_All	caspase recruitment domain-containing protein 6	−3.34	0.099
Unigene28805_All	cell death activator CIDE3	3.02	8.11
CL15195.Contig1_All	growth arrest and DNA damage-inducible protein GADD45 gamma	−2.37	0.19
CL7557.Contig1_All	immuno responsive 1	2.23	4.69
Unigene51071_All	protein AF1q	3.70	13.00
Unigene59757_All	fos-related antigen 2	3.50	11.31
Unigene47069_All	transcription factor MafB	3.48	11.16
CL15291.Contig1_All	L-amino-acid oxidase	2.54	5.82
Unigene59541_All	cathepsin S	−2.0	0.25
CL3515.Contig4_All	dendritic cell-specific-transmembrane protein	−3.59	0.083

Mannose receptor (MR), belonging to C-type lectin family, is also a class of PRRs. MR is a macrophage surface receptor that recognizes surface polysaccharides of a wide range of Gram-negative and Gram-positive bacteria, yeast, parasites and mycobacteria^35^. MR expression is modulated by immunoglobulin receptors, cytokines, pathogens and their products, which suggests that the expression of the MR may correlates with macrophage activation^36^. The MR is a 180 kDa transmembrane protein that has five domains: the cysteine-rich region, the fibronectin type II repeat domain, the eight tandem lectin-like carbohydrate recognition domains (CRDs), the transmembrane domain and the cytoplasmic carboxy-terminal domain^36^. The recognition of mannose and fucose is restricted to the CRDs, and the degree of homology between mouse and human CRDs ranges is 76–92%^36,37^. The cooperativity of the MR with TLRs has recently been described for variety of human pathogens^35^. MR also plays an important role in adaptive immunity, including antigen processing^36^. The role of MR has been described extensively in mammals, but rarely in fish. Here, two types of mannose receptor transcripts (C-type mannose receptor 1 and 2) were identified in fish^18,38^. The C-type mannose receptors 1 and 2 of large yellow croaker also have been characterized, which are up-regulated following bacterial infection^38^. DGE analysis showed that MR1 and 2 of hybrid sturgeon are up-regulated (Table 7). These results suggest mannose receptor pathway is activated in response to *Aeromonas hydrophila* infection in hybrid sturgeon. It is be interesting to follow the relationship between MR and TLRs expression following bacterial infection.

The class A scavenger receptor (cA-SR) is another important class of PRRs, and can recognize low-density lipoproteins and bacteria^39,40^. These receptors are extracellular glycoproteins, which mediate phagocytosis of negatively charged ligands^41^. The cA-SR family consists of five members: the scavenger receptor class A (SR-A), macrophage-associated receptor with collagenous structure (MARCO), scavenger receptor class A member 3 (SCARA3), scavenger receptor class A member 4 (SCARA4) and scavenger receptor class A member 5 (SCARA5). SR-A, MARCO and SCARA5 possess a scavenger receptor cysteine-rich (SRCR) domain, while SCARA3 and SCARA4 lack the domain. MARCO and SCARA3/5 were discovered in Chinese sturgeon^18^. Using DGE analysis, it found that eight MARCOs and three cA-SRs contained SRCR domain were up-regulated, which indicated the activation of the class A scavenger receptor pathway (Table 7). Interestingly, the DGE analysis showed that “phagosome” pathway, which is the downstream of scavenger receptor pathway, is the most induced pathway associated with *Aeromonas hydrophila* defense with 27 DGEs up-regulated and 30 DGEs down-regulated. The protein identification and concrete expression profile analysis of these 57 genes is shown in Table 4. The functions of phagosome are involved in bacteria degradation, pathogens killing and the regulated processing of their proteins for antigen presentation^24^. These results suggest that the hybrid sturgeon cA-SRs could recognize bacteria, which then activated phagocytosis to defense against *Aeromonas hydrophila* infection including bacteria degradation and antigen presentation for further immune response. The SRCR domain may have a potential role in bacteria recognition, which is yet to be determined. These results could provide new insight into hybrid sturgeon anti-bacterial immunity. To clarify the functions of this pathway, other components need to be identified, and the interaction among these components needs to be explored as soon as possible.

### Complement system

The complement C is an important part of innate immune system, which facilitates the ability of antibodies and phagocytic cells to clear pathogens. The mammalian complement system has three different activation pathways which include classical, alternative and mannose-binding lectin. Activation of the classical pathway is triggered by C1 that binding to antibodies antigen complex on the surface of bacterial. The C1 complex consists of C1q, C1r and C1s, which are also found in Chinese sturgeon^18,42^. The lectin pathway is initiated by binding mannose-binding lectins (MBL), associated with MBL-associated serine proteases (MASP) to an array of carbohydrate groups on the surface of bacterial^42^. The alternative pathway is triggered spontaneously, and primarily depends on recognition of host-associated molecular patterns (HAMPs)^43^. In this study, C1q (components of classical pathway), complement factor B and D (component of the alternative pathway) were all up-regulated (Table 7), which suggest activation of classical pathway and alternative complement pathway following *Aeromonas hydrophila* infection. We also identified the complement receptor (CR), both C1q and CR were up-regulated in hybrid sturgeon following *Aeromonas hydrophila* infection (Table 7). This result was different from *Yersinia*. *ruckeri* infection of Amur sturgeon^19^.

### Inflammatory cytokines and receptors

Cytokines, which are cell-signaling proteins involved in many physiological processes including the regulation of immune and inflammatory responses, could be divided into interferons (IFNs), interleukins (ILs), tumor necrosis factors (TNFs), colony stimulating factors and chemokines^44^. Interleukins (IL-1β, 6, 8, 11, 12β) and interleukin receptors (IL-1R2) were up-regulated after *Aeromonas hydrophila* infection, which indicated that the sturgeon employed these interleukins to defense against the infection. Chemokines are divided into four major subfamilies in mammals: CC, CXC, CX3C, and C, while only two groups (CC and CXC) have been identified in most teleost species^45^. CC and CXC chemokines (CCL3, CCL14, CCL19, CCL20, CCL21, CCL28, SCYA118, CXCL3, CXCL9–12, CXC14, and CXCB2) were identified in Chinese sturgeon^18^. CCL19, CCL21 and IL8 (also known as CXCL8) were up-regulated and CCL13 and CXC chemokine receptor 4 (CXCR4) were down-regulated in *Yersinia. ruckeri*- infected Amur sturgeon^19,26^. In this study, CCL4, CCL19 and IL8 were up-regulated while CXCL10, CXCR1 and CCR5 were down-regulated, suggesting the complex interaction of chemokine signaling pathway response to *Aeromonas hydrophila* infection of hybrid sturgeon. It is interesting to note that, the haematopoietic cytokines angiopoietin-1^46^ was down-regulated and matrix metalloproteinase-10, 13, 19, 28 (MMP-10, 13, 19, 28) which is critical for the vessel formation^47,48^ were up regulated. The similar results were observed in *Flavobacterium columnare* infected Mandarin fish^17^. These results imply the potential relationship between haematopoietic and vessel formation in *Aeromonas hydrophila* infection.

### T-cell and B-cell antigen activation

T lymphocytes and B lymphocytes are the main cellular components of the adaptive immune response. Fish adaptive immunity is relative primitive due to limited immunoglobulins and secondary lymphoid tissue necessary for adaptive immunity^49^. Recent, T and B cells receptors (TCR, BCR, CD3, CD4, and CD8), antigen restriction molecules (MHC I, MHC II, and DC-SIGN/CD209), co-stimulatory factors (CD80/86, CD83, CD154, and CD40) and immunoglobulins (IgM, IgD, and IgZ/T) have been identified in teleost fish, which provided evidence for the existence of adaptive immunity in fish^21^. In Chinese sturgeon, TCRα/β/γ/δ, CD3ε/ζ, CD4, and CD8, CD40, CD83, and CD80/86-CD28/CTLA4, IgM, IgD and IgL were discovered^18^. The TCR, BCR, CD4, CD8 and immunoglobulins were rearranged under the RAG1 and RAG2 regulation during T cell and B cell development^50^. DGE analysis demonstrated that RAG1, TCRδ, TCRγ, CD3, CD4, Ig light chain, Ig heavy chain were significantly down-regulated in bacterial infected sturgeon, while non-functional variable lymphocyte receptor A (VLRA) was up-regulated. These results indicate the complex interaction of adaptive immune after *Aeromonas hydrophila* infection of hybrid sturgeon.

### Antigen processing and regulators

Antigen processing is an immunological process that prepares antigens for presentation to T cells of the immune system^19^. The key components of antigen processing and regulation were present, such as class I and class II MHC molecules, integrin α/β, TNF receptor (TNR). The CD8^+^ T cells recognize protein-derived peptides in association with MHC class I (MHC-I) molecules, whereas CD4^+^ T cells recognize peptides bound to MHC class II (MHC-II) molecules^51^. MHC class I was down-regulated after *Yersinia*. *ruckeri* infection of Amur sturgeon^19^, while MHC class Ia and MHC class IIβ were up-regulated after *Aeromonas hydrophila* infection (Table 7). These results imply that the regulation of antigen processing may be different between *Aeromonas hydrophila* infected hybrid sturgeon and *Yersinia*. *ruckeri* infected of Amur sturgeon.

### Adapters, Effectors and Signal Transducers

Cell signaling is a complex system of communication that regulates basic cellular activities and coordinates cell actions. Hundreds of proteins are involved in the many signaling pathways, some of them are important adaptors, effectors and signal transducers^17^. The key components of adapters, effectors and signal transducers of anti-bacterial immune pathways were present, such as TNF receptor-associated factor (TRAF), calmodulin and nuclear factor κB (NF κB). TRAF and NF κB are the adaptor protein, effectors and transcription factors in TLR pathway^21^. The calmodulin is versatile messenger that transduces calcium signals by binding calcium ions, and mediates many crucial processes in the immune responses during the infection, such as inflammation and apoptosis^52^. During the hybrid sturgeon was infected with *Aeromonas hydrophila*, these three genes were up-regulated, implying the triggering of TLRs and calcium signals.

### Other Genes Related to Immune Response

The heat shock proteins (Hsps) play a role in both innate and adaptive immunity, including (1) elicit T-cell response specific against antigenic peptide they chaperone; (2) modulate innate response that are independent of chaperoned peptides^53^. In the present study, down-regulation of Hsp70 was similar to *Yersinia*. *ruckeri* infected of Amur sturgeon19, indicating that Hsp might be regulated by bacterial pathogens. It is noticeable that the expression of hepcidin, ferritin and serotransferrin were up-regulated following *Aeromonas hydrophila* infection, these three genes were associated with iron transport. Iron is an essential element for the growth of fish and bacterial species. Bacteria snatch the iron from their hosts, while the hosts inhibit the bacteria growth by limiting their usage of iron^54^. Bacteria have evolved many strategies to compete iron with hosts, including releasing iron-binding molecules and scavenging iron from hemoglobin and transferring^54^. The similar hepcidin up-regulation was also observed in *Flavobacterium columnare* infected Mandarin fish, which suggest that the fish produced high level of hepcidin to decrease iron level and limited the bacterial infection^17^.

## Conclusions

Based on the transcriptome sequencing results in the present study, 213 DEGs from spleen of bacterial infected hybrid sturgeon were found to be associated with pattern recognition proteins, complement system, inflammatory cytokines and receptors, antigen presenting and regulators, adapters, effectors and signal transducers and other genes related to immune cell response. Selected genes are also verified by RT-PCR. TLRs pathway, NF-kappa B pathway, class A scavenger receptor pathway, phagocytosis pathway, mannose receptor pathway and complement pathway were up-regulated in *Aeromonas hydrophila* infected hybrid sturgeon. Moreover, SSRs and SNPs were identified in hybrid sturgeon spleen transcriptome, which would be helpful for genetic linkage and QTL analysis. This study shed significant light on the anti-bacterial immune system of hybrid sturgeon, and which could lead to better understanding of interactions between the pathogen and host.

## Materials and Methods

### Ethics statement

All the fish handling and experimental procedures were approved by the Animal Care and Use Committee of the Yangtze River Fisheries Research Institute, Chinese Academy of Fishery Sciences. And all experiments were performed in accordance with relevant guidelines and regulations.

### Animals and experimental infection

Healthy hybrid sturgeons, 14–17 cm in length, were obtained from Taihu Farming Station, Yangtze River Fisheries Research Institute, Chinese Academy of Fisheries Science. The genotype of the hybrid sturgeons were validated previously^2^. All fish were acclimatized in oxygenated water at 22 ± 1 °C for a week. For experimental infection, *Aeromonas hydrophila* was cultured and suspended in sterile Dulbecco’s phosphate-buffered saline (DPBS, Sigma, USA) with the final pathogens concentration of 1.7 × 10^8^ cfu/ml. Ten hybrid sturgeons in infected group were injected intraperitoneally (i.p.) with 0.1 ml of bacterial suspension (1.7 × 10^8^ cfu/ml). Ten fish in mock infected group were injected (i.p.) with an equal volume of sterile DPBS. All the fish were returned into water tank after injection. At 7 h post-injection, the fish were anaesthetized by 0.05% MS-222 (Sigma, USA) when the infected sturgeons showed the symptom of abnormal swimming, ascites and cloacal hemorrhaging. The spleen from ten individual sturgeon from each group were collected and kept in liquid nitrogen for RNA extraction. The whole experiment was repeated twice, and two sets of bacterial infected spleens and the other two sets of mock infected spleens were collected. And they sequenced in separate lanes.

### Total RNA isolation and cDNA library construction

Total RNA was isolated from spleen tissue using Trizol Reagent (Invitrogen, USA), and genomic DNA was removed by RNase-free DNaes I (Qiagen, German). These four RNA samples (two sets of bacterial infected and the other two sets of mock infected spleens) were sent to Beijing Genomics Institute-Shenzhen (BGI, Shenzhen, China) for the Illumina deep sequencing. RNA degradation and contamination were detected by 1.5% (w/v) agarose gels. The quality and quantity of RNA were measured using Agilent 2100 Bioanalyzer (Agilent Technologies, USA). To avoid amplification of the RNA samples, the amount of total RNA for each sample used was kept at 4.0 μg with the RNA integrity number (RIN) >8.0. Poly-A-containing mRNA was further sorted by oligo-dT-attached magnetic beads and fragmented into small pieces using divalent cations under elevated temperature. Cleaved RNA fragments were converted into first-strand cDNA using reverse transcriptase and random primers, followed by second-strand cDNA synthesis using DNA polymerase I and RNase H. After the end repair process and adapter ligation step, the products were purified and amplified to generate the final cDNA libraries using the TruSeq RNA sample preparation kit.

### Illumina sequencing and transcriptome annotation

RNA deep sequencing was conducted using Illumina Hiseq2000 (paired reads, 90 bp). Raw reads were first cleaned by removing adaptor sequences and low quality sequences (Q > 20) using the Filter_fq program. The clean reads were assembled into contigs firstly, and then assembled into unigenes using the Trinity software as described for de novo transcriptome assembles without reference genome^55^.

For homology annotation, the unigenes were compared with the NCBI non-redundant protein (Nr) database using the BLASTx algorithm, with a cut-off E value of ≤10^−5^. Meanwhile, the unigenes were compared with non-redundant nucleotide (Nt) database using the BLASTn algorithm, with a cut-off E value of ≤10^−5^. Gene Ontology (GO) terms were extracted from the best hits obtained from the BLASTx against the Nr database using Blast2GO^56^. These results were then sorted by GO categories using in-house Perl scripts. BLASTx was also used to align unique sequences to the Swiss-Prot database, Clusters of Orthologous Groups (COG) and Kyoto Encyclopedia of Genes and Genomes (KEGG) (with the E value of 10^−5^) to predict possible functional classifications and molecular pathways^57^.

### Differently expressed genes identification

To identify the differential expression between infected and mock infected groups, fragments per kilobase of transcript per million fragments sequenced (FPKM) were used to normalized the gene expression levels^58^. The differential expression was analyzed by RSEM^59^ and edgeR softwares^60^. For each gene, the *p* value was computed, and then Benjamini–Hochberg false discovery rate (FDR) was applied to correct the results for *p* value. The transcripts that were increased or decreased at an estimated absolute log_2_ -fold change of ≥1 and FDR adjusted *p* value ≤ 0.001 were considered to be differently expressed. Then, GO and KEGG pathway enrichment of differential unigenes were analyzed. The repeated samples were analyzed and merged into one result by NOIseq software.

### Detection of *SSRs* and *SNPs*

MicroSAtellite (MISA) (http://pgrc.ipk-gatersleben.de/misa/misa.html) was used to analyze the microsatellite (SSR) distribution^61^. The minimum number of repeats for SSR detection was six for di-nucleotide and five repeats for tri-, quad-, penta-, and hexa-nucleotides. Potential single nucleotide polymorphisms (SNPs) were detected using SAMtools^62^ and VarScan^63^ with the following criteria: (1) total coverage and the number of reads to cover a candidate SNP (>8 reads); (2) the base quality where base calls with low Phred quality (<25) were removed from the coverage; (3) frequency of mutated bases higher than 30% among all reads covering the position.

### Quantitative real-time PCR analysis

To validate the RNA-seq differential expression studies, ten genes differently expressed between the infected and mock infected groups, including Toll like receptor 5 (TLR5), complement C1q (C1), interleukin-1 beta (IL-1β), MHC class Ia chain (MHC Ia), MHC class II beta chain (MHC IIβ), cathelicidin-OH antimicrobial peptide (CAMP), ferritin, cell death activator-3 (CIDE-3), cathepsinS and heat shock protein 70a (Hsp70a) were selected for RT-qPCR assay, using the same RNA sample used for the RNA-seq sequencing. Beta-actin (β-actin) was included as an internal reference gene to normalize the variations of input total cDNA template among samples. Briefly, total RNA was converted into first-strand cDNA using the RevertAid First Strand cDNA Synthesis Kit (Fermentas, Thermo Fisher Scientific, USA) with oligo-dT primers. Real-time PCR amplification reactions were carried out in a final volume of 20 μ l, which contained 10 μl 2 × SYBR Green PCR Master Mix (Toyobo, Japan), 1 μl diluted cDNA template, and 0.4 nM each of the forward and reverse primers. PCR amplification was performed under the following: 95 °C for 30 s, 95 °C for 15 s, 60 °C for 20 s and 72 °C for 35 s; steps 2–4 were repeated for 40 cycles^64^. All primers used for real-time PCR are listed in Table 6. Each sample was analyzed in triplicates. The relative expression level of target genes was measured with the 2^−ΔΔCT^ method and normalized by the median expression of β-actin. Results are displayed as means ± S.D. and were compared using an unpaired sample *t*-test, a p ≤ 0.05 was considered to be significant.

## Electronic supplementary material

Supplementary Dataset 1
